# Lumbar artery rupture causing hemorrhagic shock after vertebroplasty in a nonagenarian: A case report

**DOI:** 10.1097/MD.0000000000045207

**Published:** 2025-10-24

**Authors:** Shengrong Xie, Yan Wang, Yingchun Dong, Zhanyu Chen

**Affiliations:** a Department of Orthopaedics, West China Longquan Hospital Sichuan University, Chengdu, China; b Department of Breast Surgery, Longquanyi District of Chengdu Maternity and Child Health Care Hospital, Chengdu, China.

**Keywords:** case report, lumbar artery rupture, nonagenarian, retroperitoneal hematoma, vertebroplasty

## Abstract

**Rationale:**

Percutaneous vertebroplasty (PVP) is an effective and minimally invasive treatment for osteoporotic vertebral compression fractures. Although generally safe, rare but life-threatening vascular complications may occur, particularly in frail elderly patients.

**Patient concerns:**

A 90-year-old woman with a history of chronic obstructive pulmonary disease, cardiac insufficiency, and type II respiratory failure presented with severe thoracolumbar pain refractory to conservative treatment.

**Diagnoses:**

Imaging confirmed T10 and L1 osteoporotic compression fractures with severe osteoporosis (T-score − 4.5). Two hours after PVP, she developed nausea and diarrhea, followed by hypotension (73/40 mm Hg) and hemoglobin decline (Δ45 g/L). Computed tomography angiography confirmed retroperitoneal hematoma due to lumbar artery rupture.

**Interventions:**

The patient underwent urgent superselective embolization via digital subtraction angiography and transfusion therapy, followed by intensive care monitoring.

**Outcomes:**

Hemostasis was successfully achieved. She was discharged on postoperative day 14, with recovery to baseline activities of daily living at the 3-month follow-up.

**Lessons:**

This case highlights that fracture-induced displacement of lumbar arteries and transverse process hypoplasia increase vascular injury risk during PVP, atypical gastrointestinal symptoms may serve as early warning signs of retroperitoneal hemorrhage, and tailored preoperative vascular imaging and staged surgical strategies should be considered in frail patients undergoing multilevel PVP.

## 1. Introduction

The aging global population has made osteoporotic vertebral compression fracture (OVCF) a critical health concern impacting nonagenarians’ quality of life.^[1]^ Percutaneous vertebroplasty (PVP), recognized for its rapid pain relief and fracture stabilization, remains a cornerstone treatment for OVCF.^[2,3]^ However, extreme age-related anatomical alterations (severe osteoporosis with vertebral height loss) and multisystem comorbidities substantially increase perioperative risks.^[4]^ Current literature has provided limited insights into iatrogenic lumbar/segmental artery injuries during PVP.^[5]^ Timely identification of postoperative complications in geriatric patients poses diagnostic dilemmas due to 2 primary mechanisms. Primarily, diminished hemodynamic reserve in this population predisposes patients to an accelerated transition from compensated shock to circulatory collapse. Furthermore, characteristic manifestations of retroperitoneal bleeding (e.g., flank pain and hypotension) are frequently obscured by baseline cardiopulmonary comorbidities, whereas nonspecific gastrointestinal disturbances, particularly nausea and diarrhea, are often erroneously attributed to perioperative pharmacological interventions, which may result in critical delays in hemorrhage recognition and management.^[6]^

This report describes a rare case of lumbar artery rupture with hemorrhagic shock after PVP in a 90-year-old woman. We emphasize anatomical variations, atypical symptomatology, and perioperative strategies relevant to high-risk elderly patients.

## 2. Case report

### 2.1. Clinical presentation

**Figure 1.**
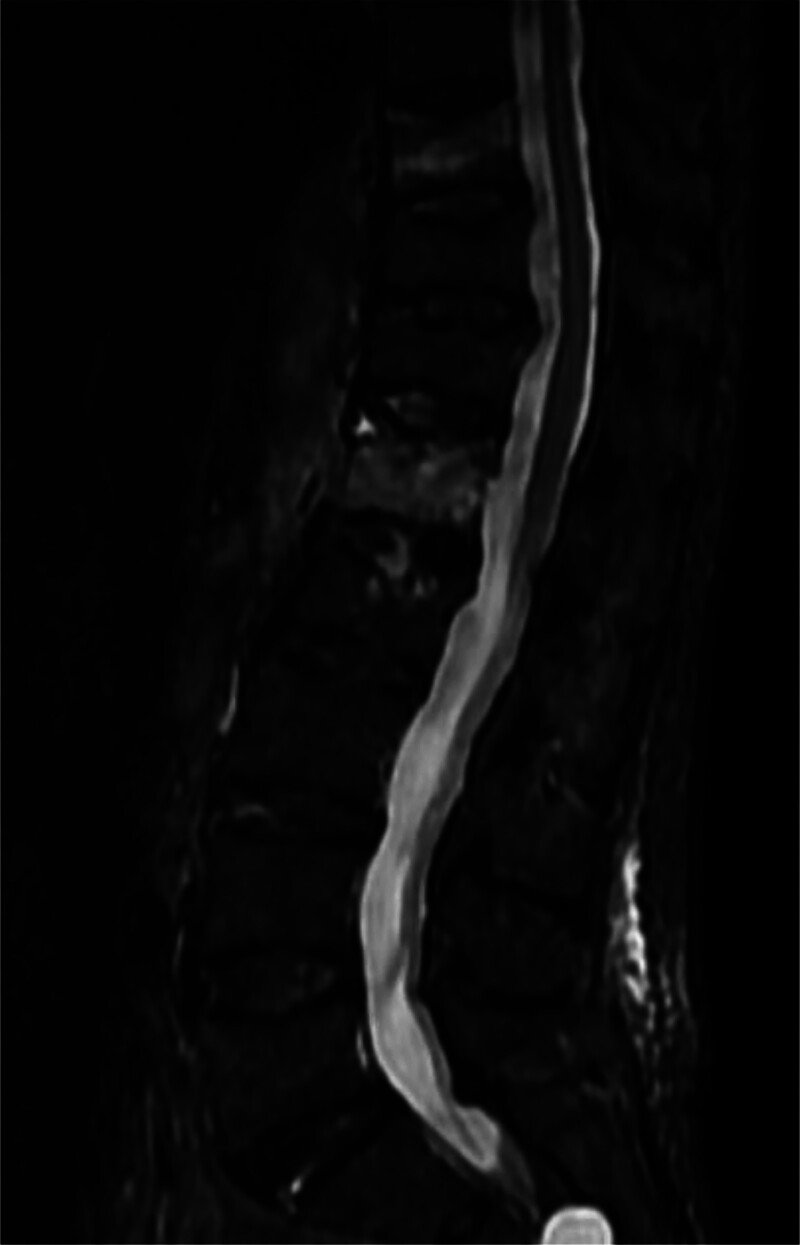
Preoperative MRI demonstrating T10 and L1 vertebral compression fractures.

**Figure 2.**
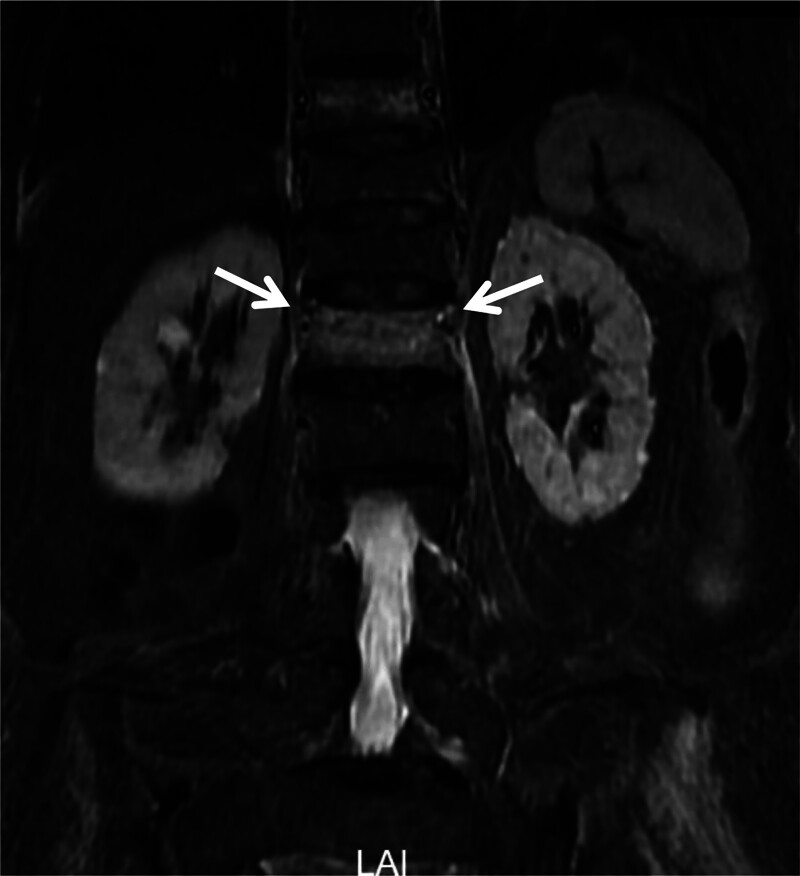
The arrow points to the L1 segmental artery, which is displaced cranially into the upper quarter of the vertebral body on this coronal MRI—a region contrary to the traditional “safe window” of the posterosuperior third.

A 90-year-old female presented with a 10-day history of refractory thoracolumbar pain (visual analog scale score 7/10) following minor trauma that was unresponsive to conservative management. The patient’s medical history included hypertension, well-controlled type 2 diabetes (HbA1c 6.3%), chronic obstructive pulmonary disease (COPD), and cor pulmonale. Physical examination revealed thoracolumbar kyphosis with localized tenderness over the T10-L1 spinous processes. Dual-energy X-ray absorptiometry demonstrated severe osteoporosis (lumbar spine T-score ‐4.5). Radiological evaluation confirmed T10 and L1 vertebral compression fractures (genant grade I) with intact posterior walls (Figs. 1 and 2). The final diagnoses were as follows:

- T10/L1 osteoporotic vertebral compression fracture.
- Primary hypertension.
- NYHA class II cardiac insufficiency.
- Type 2 diabetes mellitus.
- COPD with type II respiratory failure.

### 2.2. Multidisciplinary optimization

Preoperative stabilization was achieved through a coordinated intervention.

- Cardiology: Adjusted antihypertensive regimen (target BP < 140/90 mm Hg).
- Pulmonology: Initiated budesonide inhalation and supplemental oxygen (2 L/min via nasal cannula).
- Rehabilitation: Inspiratory muscle training.

The preoperative optimized parameters were as follows:

- SpO_2_ ≥ 96% (FiO_2_ 28%).
- Blood pressure range: 122–136/62–86 mm Hg.
- BNP level: 245 pg/mL.

### 2.3. Surgical technique

**Figure 3.**
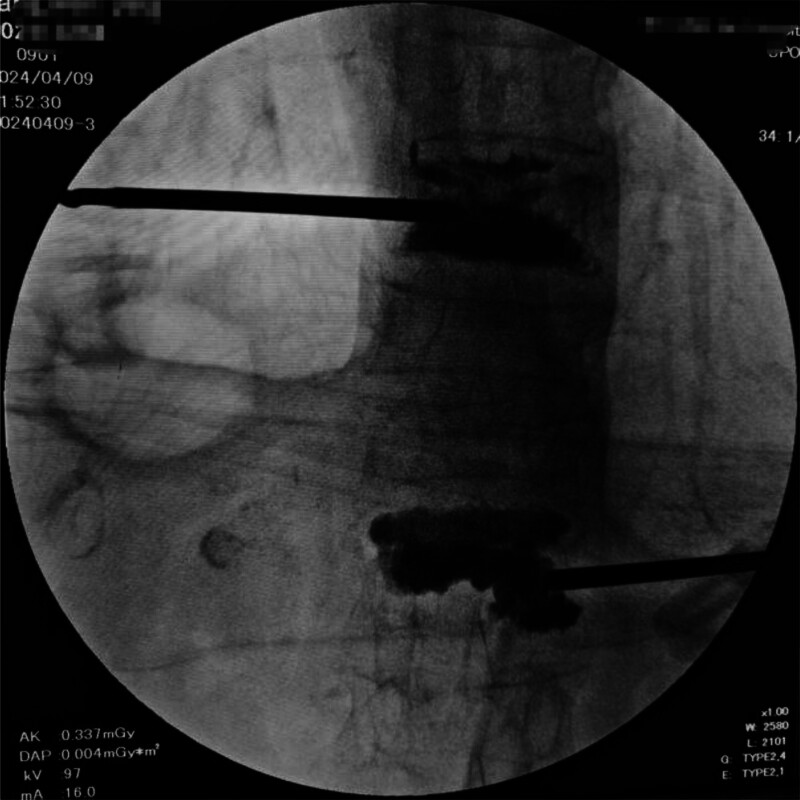
Postoperative anteroposterior view showing cement distribution in T10 and L1 vertebrae following PVP. PVP = percutaneous vertebroplasty.

**Figure 4.**
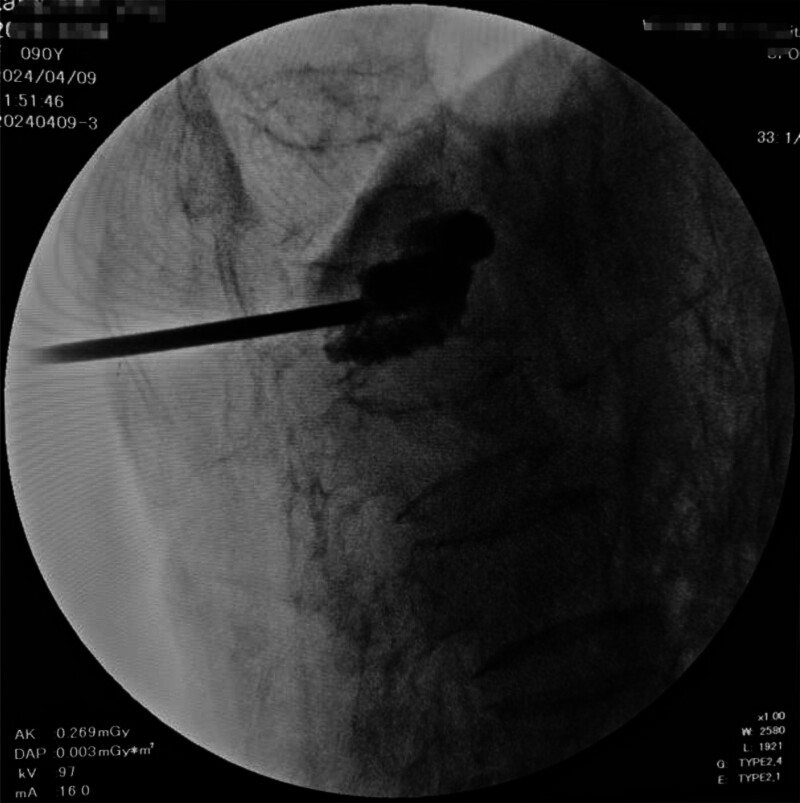
Lateral fluoroscopic view of L1 vertebral cement placement post-PVP. PVP = percutaneous vertebroplasty.

Under local anesthesia, unilateral transpedicular PVP was performed under intermittent fluoroscopic guidance. A 13-gauge trocar needle was advanced under biplanar fluoroscopy at a 20° medial trajectory. Cement injection was performed at 50 psi pressure. The cement volume was precisely administered as follows:

- T10: 6.5 mL of polymethylmethacrylate.
- L1: 7.5 mL polymethylmethacrylate.

(Figs. 3 and 4 demonstrate optimal cement distribution within the anterior third of vertebral bodies).

Intraoperative challenges included the following:

- Limited prone position tolerance (procedure duration, 50 minutes).
- Frequent involuntary torso movements during needle placement.

No immediate complications were observed, with stable vital signs maintained throughout.

### 2.4. Postoperative course

#### Two hours post-op

Developed nausea and diarrhea, partially alleviated by intravenous omeprazole 40 mg.

#### Five hours post-op

Acute deterioration manifested as lethargy (GCS 12), hypotension (73/40 mm Hg), and a hemoglobin drop from 122 to 77 g/L (Δ45 g/L).

### 2.5. Diagnostic imaging

**Figure 5.**
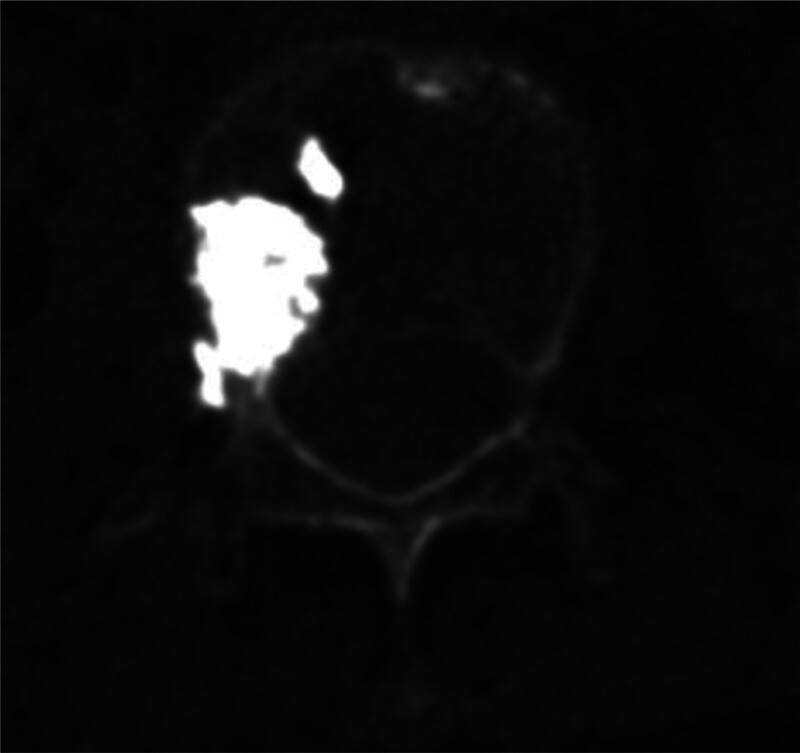
Postoperative CT scan confirming right L1 transverse process fracture along the needle trajectory.

**Figure 6.**
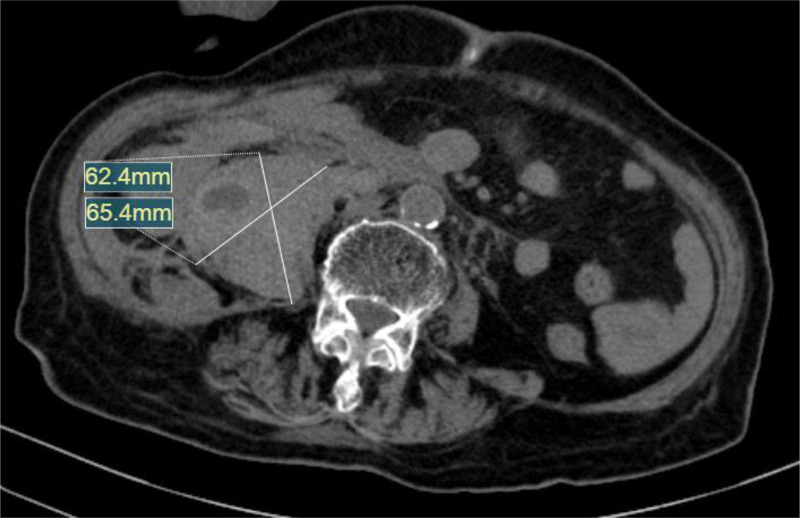
Abdominal CT reconstruction illustrating retroperitoneal hematoma (6.24 × 6.24 cm).

**Figure 7.**
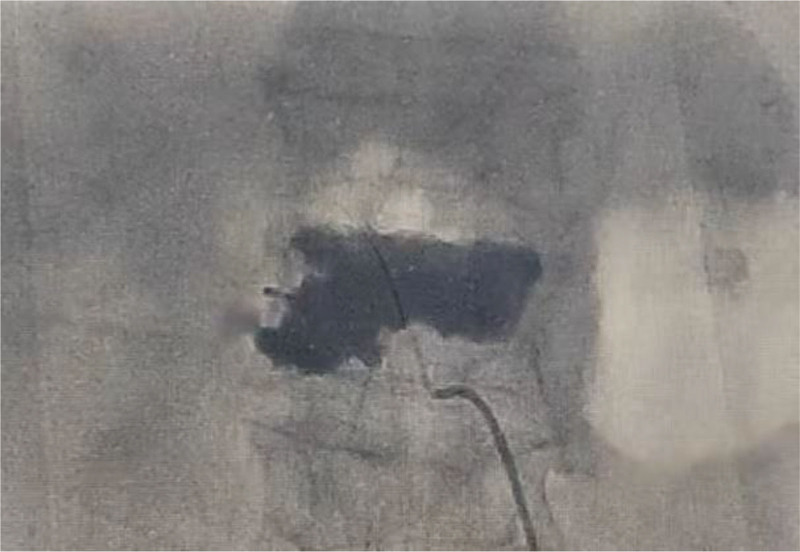
Pre-contrast digital subtraction angiography (DSA) of the lumbar arteries.

**Figure 8.**
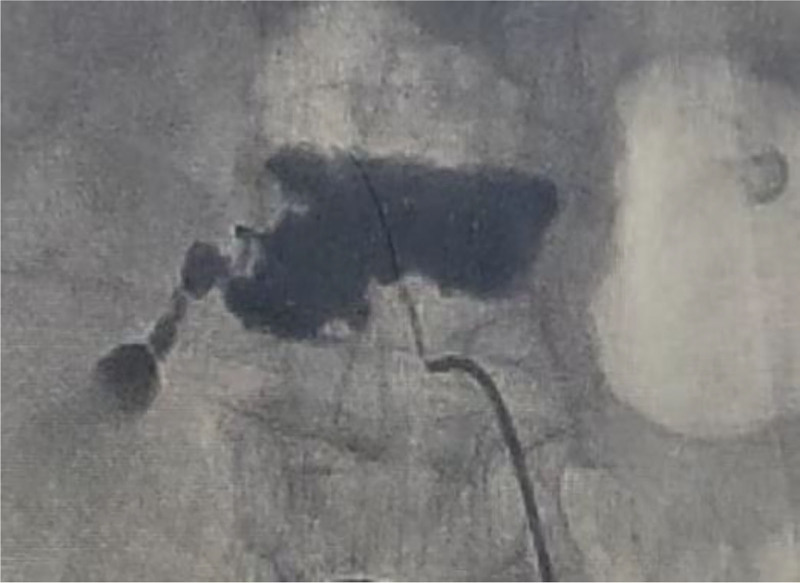
Post-contrast DSA demonstrating active extravasation from the ruptured L1 lumbar artery (white arrow). DSA = digital subtraction angiography.

Computed tomography (CT) imaging revealed a fracture of the right L1 transverse process consistent with the needle trajectory, accompanied by a sizable retroperitoneal hematoma measuring 12.2 × 6.24 × 6.24 cm (Figs. 5 and 6). Subsequent digital subtraction angiography confirmed active contrast extravasation originating from the distal segment of the right L1 lumbar artery, indicating active arterial bleeding (Figs. 7 and 8).

### 2.6. Intervention

The patient underwent emergency superselective embolization using 500 to 700 μm gelatin sponge particles to achieve hemostasis. This was followed by transfusion of 5 units of packed red blood cells to address hemorrhagic shock and maintain hemodynamic stability. Postprocedurally, the patient was admitted to the intensive care unit for 48 hours of close monitoring and supportive management.

### 2.7. Outcomes

The patient was discharged on postoperative day 14 with full ambulation. At the 3-month follow-up, functional assessment revealed complete recovery of activities of daily living scores, returning to the preoperative baseline.

### 2.8. Patient perspective

The patient and her family expressed satisfaction with the treatment outcome and emphasized the importance of timely recognition and management of complications.

## 3. Discussion

### 3.1. Procedural risks in geriatric patients

PVP remains the first-line intervention for refractory OVCF because of its minimally invasive nature and rapid analgesic efficacy.^[7–9]^ While existing evidence supports its safety profile in nonagenarians,^[10]^ this population presents unique challenges: multisystem comorbidities (particularly cardiopulmonary and cognitive impairments) amplify perioperative risks^[11]^; and limited prone position tolerance—exacerbated in COPD patients with hyperinflated lungs and dementia-related agitation.^[12,13]^ Recent studies have underscored that nonagenarians undergoing vertebroplasty are more likely to experience systemic complications, including cardiopulmonary decompensation and postoperative delirium, than younger cohorts.^[14,15]^ However, reports of vascular injury in this age group remain exceedingly rare.

Our case exemplifies these challenges: despite multidisciplinary preoperative optimization, intraoperative torso instability (attributable to COPD-induced dyspnea, cor pulmonale, and presbycusis-related disorientation) likely contributed to accidental vascular penetration. This observation further underscores the importance of implementing a staged surgical strategy for patients requiring multisegment PVP who present with suboptimal baseline health status. Such an approach may strategically reduce the prone positioning duration per session, enhance postural compliance, and mitigate procedural interference caused by intraoperative patient movement.

### 3.2. Fracture-induced vascular anatomical shifts

Conventional anatomical studies position the L1 segmental arteries within the vertebral midzone.^[16]^ However, Xu et al computed tomography angiography analysis has identified a “safe window” at the posterosuperior third of intact L1 vertebrae^[17]^—a finding that is not generalizable to compressed OVCF vertebrae. Preoperative coronal MRI revealed cranial migration of the L1 segmental artery to the upper quarter (Fig. 2), suggesting fracture-induced vascular displacement. This mandates preoperative vascular mapping (via MRI or computed tomography angiography) of deformed vertebrae to redefine the surgical trajectories.

### 3.3. Technical limitations of fluoroscopic guidance

**Figure 9.**
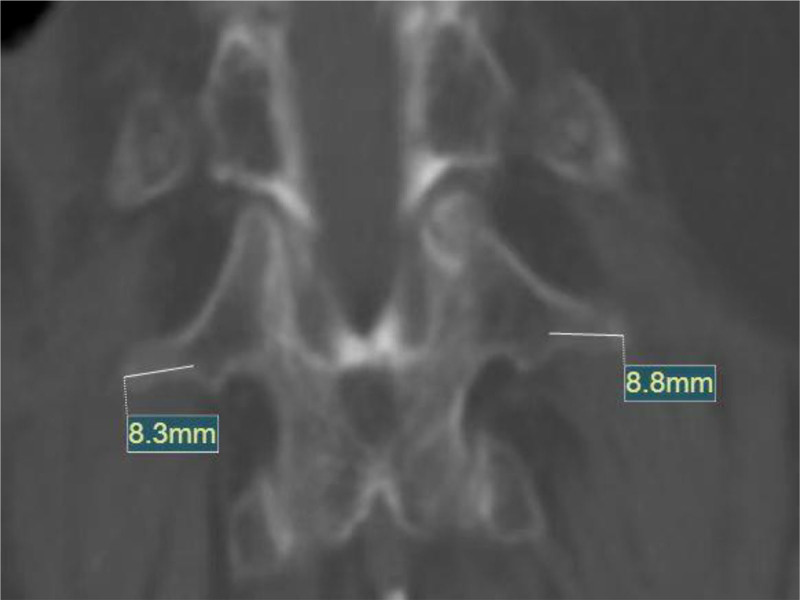
Preoperative CT reconstruction revealed lengths of 8.3 mm for the right L1 transverse process and 8.8 mm for the left.

The L1 vertebra exhibits a distinct anatomical feature—its transverse processes are notably smaller and more hypoplastic than those in other lumbar segments. Preoperative CT reconstruction in this patient confirmed that the right L1 transverse process measured only 8.3 mm in length, and the left measured 8.8 mm (Fig. 9). This reduced size increases the inherent risk of pedicular breach during needle advancement. Although C-arm fluoroscopy is well-established as the standard imaging guidance for PVP, its fundamental constraints as a two-dimensional imaging modality have been extensively documented.^[18]^ This technical limitation manifests in compromised spatial resolution and anatomical superimposition, potentially hindering precise needle trajectory planning during multisegment procedures.

In our institution, intermittent fluoroscopy is routinely employed instead of continuous real-time monitoring. This strategy aims to minimize cumulative radiation exposure for both the patient and the surgical team, particularly in elderly individuals who often require prolonged operative times or multilevel vertebroplasty. Published data indicate that intermittent fluoroscopy can reduce radiation dose by approximately 64% compared with continuous imaging.^[19]^ However, this technique necessitates frequent C-arm repositioning and introduces blind intervals in trajectory monitoring, which may delay the recognition of cortical breaches or vascular penetration.

Notably, intraoperative fluoroscopy failed to detect 2 clinically significant events.

1. Osteopenic penetration: Severely diminished bone density (T-score ‐4.5) resulted in attenuated tactile feedback during transpedicular advancement, allowing unintentional transverse process perforation.
2. Retrograde migration: Post-procedural CT reconstruction revealed anterior cortical violation with subsequent retroperitoneal hematoma formation, which was undetectable by standard anterior/lateral fluoroscopic views.

This case underscores a critical paradox in geriatric PVP: while intermittent fluoroscopy reduces the cumulative radiation dose (particularly relevant in multilevel procedures), it simultaneously increases vulnerability to occult vascular injuries in osteoporotic anatomy. Surgeons must recognize abrupt loss of needle resistance as a potential sentinel sign of cortical breach, which mandates immediate trajectory reassessment via oblique fluoroscopic projections or cone-beam CT when available.

### 3.4. Atypical presentations as early warning signs

Our literature review revealed only 5 reported vertebroplasty-related lumbar artery injuries (none involving L1) prior to 2018,^[5]^ with the case of Umeda et al demonstrating immediate bleeding as a cardinal sign.^[20]^ Contrary to classic triads (flank pain, hypotension, and neurologic deficits),^[5]^ our patient manifested prodromal gastrointestinal symptoms (nausea/diarrhea at 2 hours) preceding hemodynamic collapse by 3 hours, aligning with Yu et al description of enteric hyperactivity as a potential indicator of occult hemorrhage.^[21]^ Retroperitoneal hematoma may irritate the celiac plexus, triggering vagal-mediated gastrointestinal symptoms prior to overt hemodynamic compromise. This temporal sequence highlights 2 critical alerts: absence of surgical site bleeding cannot exclude vascular injury and unexplained postoperative gastrointestinal distress warrants urgent hematocrit monitoring and abdominal imaging.

In summary, the lumbar artery injury in this case resulted from the combined effects of multiple factors, including surgical approach selection, intermittent C-arm fluoroscopy, vertebral compression-induced vascular displacement, and patient-specific factors, such as advanced age, cardiopulmonary insufficiency, and poor intraoperative cooperation. In clinical practice, clinicians should fully recognize the impact of these factors, develop comprehensive surgical plans and risk prevention measures tailored to individual conditions, minimize the risk of lumbar artery injury and other complications, and ultimately ensure surgical safety and therapeutic efficacy.

## 4. Conclusion

This case demonstrates that lumbar artery rupture after PVP, though rare, can be life-threatening in frail elderly patients. Practical clinical implications include: using preoperative CT or MRI vascular mapping in cases of severe vertebral deformity or suspected transverse process hypoplasia to identify fracture-related vascular displacement; considering staged multilevel PVP in frail patients to shorten prone positioning time and reduce risks; and incorporating early detection of atypical gastrointestinal symptoms into postoperative monitoring, as these may signal retroperitoneal hemorrhage. Early diagnosis and endovascular intervention are critical for improving outcomes in this high-risk population.

## Author contributions

**Conceptualization:** Shengrong Xie, Yan Wang, Yingchun Dong, Zhanyu Chen.

**Supervision:** Yan Wang.

**Validation:** Yingchun Dong, Zhanyu Chen.

**Visualization:** Yingchun Dong.

**Writing – original draft:** Shengrong Xie.

**Writing – review & editing:** Shengrong Xie, Yan Wang.
